# Ultra-processed foods and incident cardiovascular disease and hypertension in middle-aged women

**DOI:** 10.1007/s00394-023-03297-4

**Published:** 2023-12-26

**Authors:** Anushriya Pant, Sarah Gribbin, Priscila Machado, Allison Hodge, Jason H. Wasfy, Lisa Moran, Simone Marschner, Clara K. Chow, Sarah Zaman

**Affiliations:** 1 Westmead Applied Research Centre and Faculty of Medicine and Health, University of Sydney, Westmead, NSW 2145 Australia; 2 Department of General Medicine, The Alfred Hospital, Alfred Health, Melbourne, VIC Australia; 3 Institute for Physical Activity and Nutrition, School of Exercise and Nutrition Sciences, Deakin University, Geelong, VIC Australia; 4 Cancer Epidemiology Division, Cancer Council Victoria, Melbourne, VIC Australia; 5 Centre for Epidemiology and Biostatistics, Melbourne School of Population and Global Health, The University of Melbourne, Parkville, Melbourne, VIC Australia; 6 Department of Cardiology, Westmead Hospital, Westmead, NSW Australia; 7 Cardiology Division, Massachusetts General Hospital and Harvard Medical School, Boston, MA USA; 8 Monash Centre for Health Research and Implementation, Monash University, Melbourne, VIC Australia

**Keywords:** Ultra-processed foods, Dietary intake, Sex-specific, Hypertension, Prevention

## Abstract

**Purpose:**

Ultra-processed food (UPF) intake has increased in recent decades, yet limited knowledge of long-term effects on cardiovascular health persists and sex-specific data is scant. We determined the association of UPF intake with incident cardiovascular disease (CVD) and/or hypertension in a population-based cohort of women.

**Methods:**

In the Australian Longitudinal Study on Women’s Health, women aged 50–55 years were prospectively followed (2001–2016). UPFs were identified using NOVA classification and contribution of these foods to total dietary intake by weight was estimated. Primary endpoint was incident CVD (self-reported heart disease/stroke). Secondary endpoints were self-reported hypertension, all-cause mortality, type 2 diabetes mellitus, and/or obesity. Logistic regression models assessed associations between UPF intake and incident CVD, adjusting for socio-demographic, medical comorbidities, and dietary variables.

**Results:**

We included 10,006 women (mean age 52.5 ± 1.5; mean UPF intake 26.6 ± 10.2% of total dietary intake), with 1038 (10.8%) incident CVD, 471 (4.7%) deaths, and 4204 (43.8%) hypertension cases over 15 years of follow-up. In multivariable-adjusted models, the highest [mean 42.0% total dietary intake] versus the lowest [mean 14.2% total dietary intake] quintile of UPF intake was associated with higher incident hypertension [odds ratio (OR) 1.39, 95% confidence interval (CI) 1.10–1.74; *p* = 0.005] with a linear trend (*p*_trend_ = 0.02), but not incident CVD [OR 1.22, 95% CI 0.92–1.61; *p* = 0.16] or all-cause mortality (OR 0.80, 95% CI 0.54–1.20; *p* = 0.28). Similar results were found after multiple imputations for missing values.

**Conclusion:**

In women, higher UPF intake was associated with increased hypertension, but not incident CVD. These findings may support minimising UPFs within a healthy diet for women.

**Supplementary Information:**

The online version contains supplementary material available at 10.1007/s00394-023-03297-4.

## Introduction

Cardiovascular disease (CVD) is the main cause of mortality in women [1]. Healthy dietary intake is a key aspect of primary prevention of CVD and cardiovascular risk factors. Hypertension is one of the most important modifiable risk factors for CVD and a major cause of premature death worldwide [2]. Global modernisation has brought significant changes to our current way of living, including the introduction of ultra-processed food (UPF). UPF is defined within the NOVA classification as formulations of ingredients, mostly exclusive industrial use, that result from series of industrial processes [3]. Monteiro et al. [3] first developed the NOVA classification, which categorises foods according to the degree of industrial processing. This involves methods used for food manufacturing, such as extraction, preservation, or making of ingredients [3].

UPF has become heavily advertised and marketed in both high-income and low-income countries in recent decades [4]. Being readily available and convenient, UPFs are now a major component of dietary intake worldwide [4]. In Australia, UPFs make up 42% of total energy intake [5]. However, increased UPF intake leads to lower intake of fresh, minimally processed foods [6]. Additionally, UPF intake is associated with higher intake of energy, salt, free sugars, and saturated and trans-unsaturated fat, with lower intake of fibre and cardio-protective micronutrients. This is considered to lead to hypertension, dyslipidaemia, weight gain [6, 7] and adverse health outcomes including diabetes mellitus (DM), CVD, and obesity [8–10]. To date, several studies have prospectively explored the link between UPFs and CVD [11–16]. However, sex-specific analyses are still limited only one study examined incident CVD by sex, reporting a detrimental effect of higher UPF intake in women similar to men [17]. For all-cause mortality, the only two studies that performed sex-specific analyses demonstrated significant associations with higher UPF intake in both men and women, but no sex interaction [18, 19]. A more recent study on all-cause mortality and UPF intake in a Korean population, however, found that there was no significant relationship in either men or women [20].

This is still a new and evolving research area. Increasing evidence has linked UPF intake to many chronic diseases [8–10]. However, the magnitude of the impact of UPF intake on CVD and hypertension in Australia is unclear. This limits the body of evidence available to inform national policies, including the current review of the Australian Dietary Guidelines. Presently, Australian Dietary guidelines have not specifically addressed intake of UPFs and its cardiovascular health effects. Moreover, dietary studies on cardiovascular health in women alone are limited [21, 22]. Historically, there has been general dearth of sex-specific literature in CVD and a real need for a sex-specific approach to the prevention and treatment of CVD persists.

We therefore aimed to analyse the prospective association between UPF intake and incident CVD and/or cardiovascular risk factors in women.

## Methods

The Australian Longitudinal Study on Women’s Health (ALSWH) is a prospective cohort study that was formed in 1996, following more than 57,000 Australian women over a period of 20 years [23]. Women were randomly selected from the Australian Medicare Databases from three age groups (born 1921–1926, 1946–1951, and 1973–1978). Participants provided informed consent to regular surveys and data linkage to hospital admissions, Medicare Benefits Schedule, Pharmaceutical Benefits Scheme, and the National Death Index (NDI). The study methods have been described in detail previously [22].

In the current study, women (age 50–55 years at baseline) were included from the 1946–1951 cohort. We included participants who had completed the third survey and were free of CVD. The third survey (year 2001) included the first dietary assessment and was used as the baseline for our analyses. Participants who completed surveys at baseline were followed every 3–4 years until 2016. We excluded women who reported CVD in surveys 1–3, had incomplete dietary assessment, and had implausible energy intake (< 2092 or > 14,644 kilojoules/day) [24].

### Dietary assessment

Self-reported dietary intake was collected from the third survey using a 101-item food frequency questionnaire (FFQ) (Dietary Questionnaire for Epidemiological Studies version 2) [25]. This questionnaire has been previously validated [26]. Participants were asked to report their consumption of food and beverages over the last 12 months, with responses from ‘never’ to ‘3 or more times per day'. Respondents were provided with portion size photographs to select their portion sizes for food items. UPF intake was assessed using the 74 food items and 6 beverages reported in the FFQ, identified according to the NOVA classification system (Online Resource Table 1) [3]. We categorised all food items from the FFQ into the four NOVA groups: (1) unprocessed or minimally processed foods, (2) processed culinary ingredients, (3) processed foods, and (4) ultra-processed foods [3]. All classifications were cross-checked between two independent reviewers and discrepancies were resolved with a group consensus. Food items that were difficult to discriminate were compared with National Nutrition and Physical Activity Survey (2011–2012) and for those foods where the classification was still unclear, the conservative alternative was selected, for example homemade/processed over ultra-processed [5, 27] The dietary share of UPF intake was calculated as a proportion (%) of total weight of food and beverage consumed (grams per day (g/d)). Participants were divided into quintiles of UPF intake, with the lowest consumers belonging to the first quintile and the highest consumers to the fifth. Glycaemic index (GI) and glycaemic load (GL) values were ascertained using the 2002 International Table of GI and GL values [28].

### Primary and secondary endpoints

The primary endpoint was incident CVD (physician-diagnosed self-reported heart disease or stroke). CVD was based on follow-up survey questions every 3–4 years until 2016 with women who responded, ‘Yes’ to any of the following questions, ‘In the past three years, have you been diagnosed or treated for heart disease?’ and/or ‘in the past three years, have you been diagnosed or treated for stroke?’. Secondary endpoints were incident hypertension, type 2 DM, obesity, and all-cause mortality (from the NDI). Incident hypertension, type 2 DM, and obesity were self-reported and identified at follow-up surveys every 3–4 years until 2016, defined as the first reported diagnosis in participants without the condition at baseline. Body mass index (BMI) (kg/m^2^) was calculated from self-reported weight and height, with a BMI ≥ 30 kg/m^2^ considered as obese and BMI of 25.0 to < 30 kg/m^2^ considered as overweight [29].

### Confounders

Potential socio-demographic, medical, and dietary confounders were all self-reported and determined from baseline surveys and selected based on previous literature and a Directed Acrylic Graphic. Socio-demographic variables included age (continuous), area of residence (continuous, evaluated using the Accessibility and Remoteness Index of Australia (ARIA +)), marital status (categorical), occupation (categorical), country of birth (categorical), qualification (categorical), and household income (categorical). Medical comorbidities included health conditions (type 2 DM and hypertension) (categorical), BMI (continuous), menopausal status (categorical), physical activity levels (categorical), and smoking status (categorical). Dietary variables were all continuous and included total fibre, total fat, total carbohydrate, total protein, total energy intake (kilojoules/day), and alcohol intake. Physical activity was converted into metabolic equivalents (METs): ‘sedentary’ 0–40 METs min/week, ‘low’ 40–600 METs min/week, ‘moderate’ 600–1200 METs min/week and ‘high ≥ 1200 METs min/week [30, 31].

### Statistical analysis

All statistical analyses were performed using SAS version 9.4 for Window (Cary, North Carolina: United States). For descriptive statistics, we assessed baseline characteristics for eligible women across quintiles of UPF intake as a proportion (%) of total dietary intake (g/d) using Chi-squared (*χ*^2^) tests (categorical variables) and analysis of variance (ANOVA) (continuous variables). Logistic regression models were used to assess the prospective association between UPF intake and endpoints. The first quintile (lowest UPF intake) was the reference. Variables associated with UPF intake in bivariate Pearson correlations were initially included in the final multivariate model. We excluded total saturated fat, monounsaturated fat, polyunsaturated fat, sugars, and sodium since these showed significant collinearity with other dietary variables (*r* ≥ 0.8). Additional covariates were chosen based on existing literature and univariate testing, where potential confounders were included in the models if they returned a significance level of *p* ≤ 0.05. All covariates were baseline measures.

We selected a four-model approach using knowledge from previous cohort studies [17, 18]: univariate model; model 1 adjusted for age, area of residence, marital status, occupation, country of birth, qualification, and household income; model 2 adjusted for model 1 and further controlled for BMI, menopausal status, type 2 DM, hypertension, physical activity levels, and smoking status; and model 3 adjusted for model 2 and dietary variables (total fibre, total carbohydrate, total fat intake, total protein, total energy intake, total alcohol). When calculating the p for linear trend, the independent variable (proportion of UPF intake) was treated as continuous.

We analysed participants free from CVD in all models for both primary and secondary outcomes. In our analyses with secondary outcomes of incident hypertension, type 2 DM, and obesity, we only included participants without the outcome condition at baseline.

Multiple imputations were performed for variables with high amounts of missing data (occupation, 7.6%; household income, 16.1%; physical activity, 4.1%) using chained equations (fully conditional method). Sensitivity analyses were performed to assess the associations with multiple imputation.

All tests were 2-sided, and statistical significance was set at *p* ≤ 0.05.

## Results

### Study sample

**Fig. 1:**
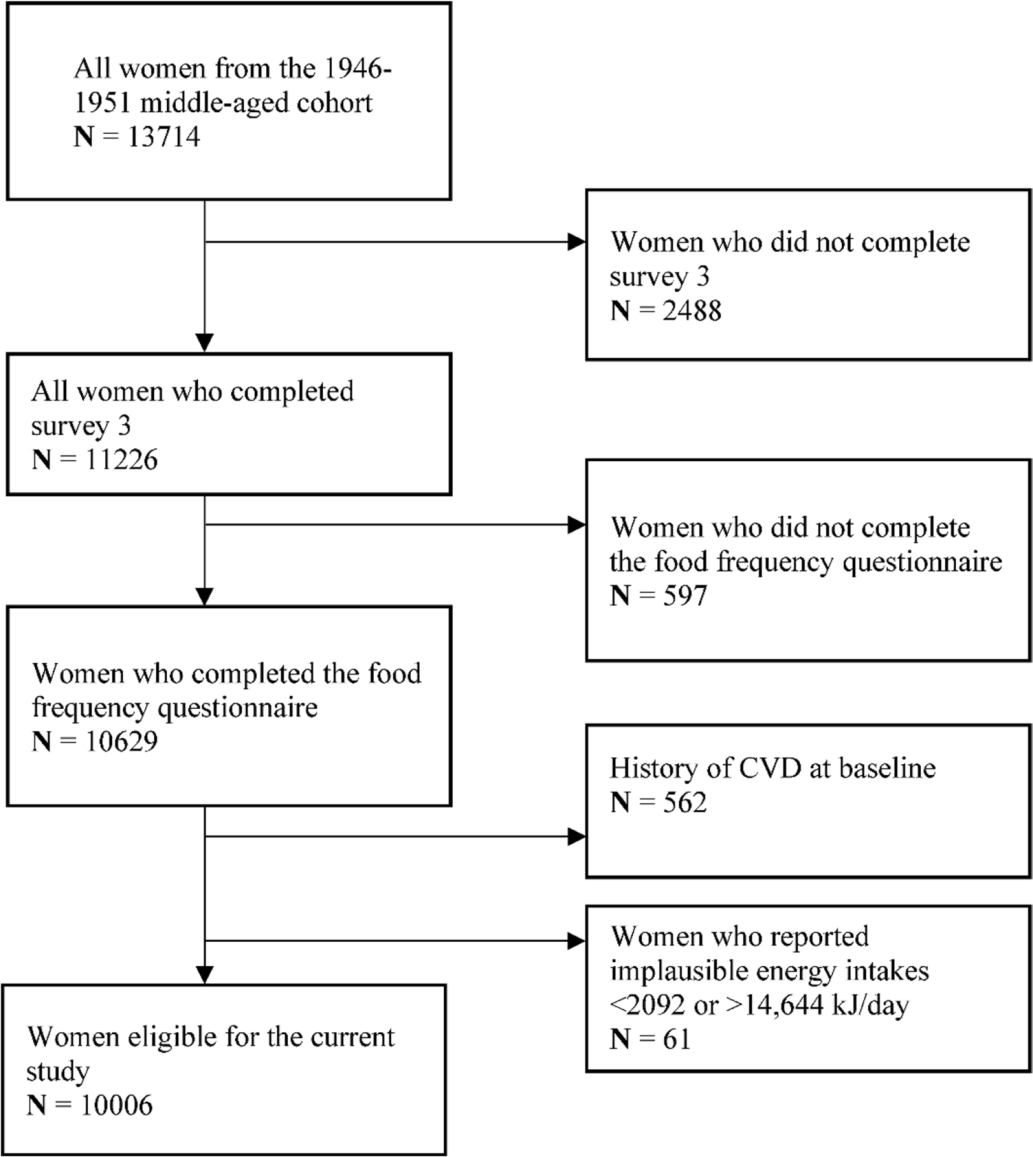
Study flow chart of identifying participants that meet the inclusion/exclusion criteria. *CVD* cardiovascular disease, *kj* kilojoules

A total of 13,714 women (1946–1951 cohort) were recruited into the ALSWH. We excluded 2489 women who did not complete survey 3 and further excluded those who had incomplete FFQs (*n* = 597), had diagnosed CVD at baseline (*n* = 562) or reported mplausible energy intake (*n* = 61). The final cohort size included 10,006 women (Fig. 1).

### Baseline characteristics

**Fig. 2:**
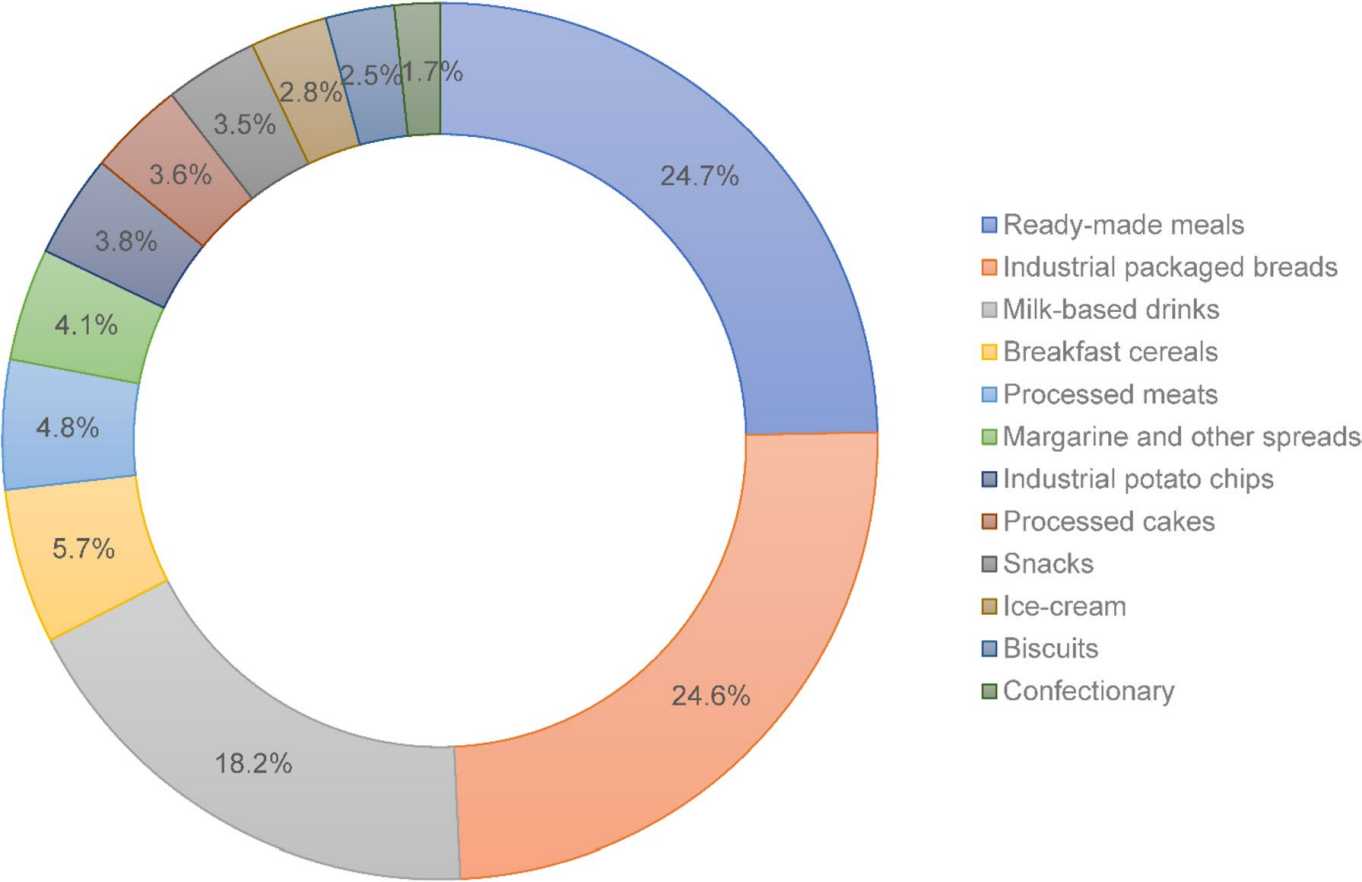
Proportion of food groups that comprised ultra-processed food intake among middle-aged Australian women

At baseline, women (mean age 52.5 ± 1.5 years, mean BMI 26.8 ± 5.4 kg/m^2^) consumed a mean of 26.6 ± 10.2% UPFs (mean 347.8 ± 181.6 g/d) as part of their total dietary intake (mean 1299.7 ± 405.5 g/d). Women in the highest quintile of UPF intake consumed a mean of 42.0 ± 7.4% total dietary intake compared to 14.2 ± 3.1% total dietary intake for those in the lowest quintile (Table 1). Frequently consumed UPF items included: ready-made meals (e.g., meat pies, hamburgers) (24.7%), packaged breads (24.6%), milk-based drinks (e.g., sweetened yoghurts, flavoured milk) (18.2%), breakfast cereals (5.7%), and processed meat (4.9%) (Fig. 2). Significant differences were seen across quintiles of UPF intake for area of residence, country of birth, marital status, type 2 DM, hypertension, BMI (continuous and categorical), smoking status, and physical activity level. Women who consumed the highest proportional intake of UPF (quintile 5) compared to those in quintile 1 (least UPF) were most likely to live in metropolitan/inner regional as, be born in Australia/Europe, have low physical activity and be separated or divorced (Table 1). These women were also most likely to have hypertension yet have normal weight and never smoke. Women with the lowest proportion of UPF intake were most likely to have type 2 DM, be widowed or married/de facto, have higher physical activity, have obesity, and currently smoke. Women in quintile 1 (lowest proportion of UPF) were most likely to be from Asia, and outer regional and remote/very remote areas. Women in quintiles 2 and 3 were also more likely to have higher BMI than those with the in quintile 1 (Table 2).

**Table 1:** Baseline characteristics of middle-aged Australian women according to ultra-processed food intake quintiles

Characteristics	Quintile 1	Quintile 2	Quintile 3	Quintile 4	Quintile 5	P value
Number of participants	2000	2001	2002	2001	2001	N/A
% Ultra-processed food intake (g/d)	14.2	20.7	25.4	30.8	42.0	< .0001
Age at baseline, mean ± SD (years)	52.5 ± 1.4	52.5 ± 1.5	52.5 ± 1.5	52.6 ± 1.5	52.5 ± 1.5	0.23
Menopausal status (%)						0.59
HRT	16.4	18.4	15.9	17.7	17.7	
OCP	2.1	2.1	2.2	2.3	2.2	
Pre-menopause	9.4	9.2	10.7	8.9	8.9	
Post-menopause	25.5	24.0	24.6	24.1	24.9	
ARIA + group (%)						0.004
Metropolitan	34.2	34.2	35.0	33.4	34.7	
Inner regional	38.2	40.7	39.8	44.1	41.9	
Outer regional	22.6	20.4	21.4	18.9	19.4	
Remote	3.7	4.2	3.1	3.0	3.2	
Very remote	1.3	0.5	0.8	0.6	0.9	
Country of birth (%)						< .0001
Australia	76.12	76.71	78.77	78.24	77.53	
Other ESB	13.83	15.02	13.4	14.13	12.78	
Europe	5.65	4.69	5.76	5.35	7.32	
Asia	3.58	2.47	1.47	1.56	1.62	
Education (%)						0.22
No formal qualifications	16.7	16.6	15.0	15.8	15.4	
School/intermediate certificate	29.1	32.9	31.9	33.2	32.8	
Higher school/leaving certificate	17.8	17.1	17.0	17.1	15.1	
Trade/apprenticeship	3.5	3.2	3.8	2.9	3.5	
Certificate/diploma	17.1	15.6	16.8	16.2	17.5	
University degree	9.3	10.0	10.1	9.9	9.9	
Higher degree (master’s, PhD)	6.5	4.6	5.5	4.9	5.8	
Occupation						0.20
No paid job	27.9	29.1	26.7	28.2	26.7	
Clerk/sales/transport	19.2	20.5	22.3	19.4	20.3	
Associate professional/advanced clerk	19.9	18.8	20.1	22.2	20.0	
Professional/manager	33.0	31.5	31.0	30.2	33.1	
Marital status (%)						0.01
Married/De facto	80.6	83.2	83.2	82.9	79.9	
Separated/divorced	13.2	10.4	11.3	11.5	13.6	
Widowed	3.1	3.2	2.9	3.4	2.7	
Household annual income ($AU) (%)						0.91
< 16,000	7.6	7.3	6.6	7.0	6.3	
16,000–51,999	50.3	50.4	51.9	50.0	52.2	
> 51,999	42.2	42.3	41.5	42.9	41.5	
Type 2 diabetes mellitus (%)	5.8	4.6	4.0	3.6	3.4	0.001
Hypertension (%)	24.7	29.6	28.0	28.9	26.1	0.003
Cancer (%)	3.1	2.9	3.0	3.2	4.0	0.30
PCOS	1.4	1.4	1.1	0.9	1.4	0.46
GDM	4.3	3.3	4.8	3.6	3.8	0.11
BMI, mean ± SD	26.5 ± 5.3	27.0 ± 5.3	27.1 ± 5.5	26.9 ± 5.5	26.3 ± 5.2	< .0001
BMI (%)						0.004
Normal weight	44.7	40.6	40.6	43.1	46.4	
Overweight	32.1	33.5	33.5	31.8	32.1	
Obese	21.7	24.5	24.6	24.2	20.0	
Smoking status (%)						0.0001
Never smoked	58.0	61.7	62.6	61.9	62.6	
Ex-smoker	24.5	23.6	23.6	25.1	25.4	
Current smoker	17.5	14.6	13.8	13.0	12.0	
Physical activity (%)						0.02
Sedentary	16.8	18.7	16.2	16.7	16.0	
Low	30.6	32.9	33.8	35.2	33.9	
Moderate	21.1	20.0	22.7	21.4	22.0	
High	31.5	28.4	27.3	26.6	28.2	

*ARIA*+  Accessibility and Remoteness Index of Australia, *BMI* body mass index, *ESB* English-speaking background, *GDM* gestational diabetes mellitus, *HRT* hormone replacement therapy, *NA* not applicable, *OCP* oral contraceptive pill, *PCOS* polycystic ovary syndrome, *SD* standard deviation

*p* values were calculated using *χ*^2^ test or analysis of variance (ANOVA)

**Table 2:** Nutrient profile of middle-aged women according to ultra-processed food intake quintiles at baseline

Nutrient breakdown	Quintile 1	Quintile 2	Quintile 3	Quintile 4	Quintile 5	*p* value
Total energy intake^a^	5926	6472	6644	6914	7028	< .0001
Total carbohydrates^b^	154.3	170.0	175.5	183.1	187.9	< .0001
Total sugars^b^	73.5	77.5	79.8	83.1	84.7	< .0001
Total sodium^c^	1827.3	2067.1	2149.9	2246.6	2255.0	< .0001
Total fat^b^	54.0	60.7	62.8	66.3	68.1	< .0001
Total monounsaturated fat^b^	19.3	21.5	22.2	23.4	23.7	< .0001
Total polyunsaturated fat^b^	7.7	9.1	9.8	10.5	12.3	< .0001
Total saturated fat^b^	21.7	24.5	25.2	26.5	26.2	< .0001
Total cholesterol^c^	241.3	244.7	239.8	243.1	232.2	0.0002
Total protein^b^	79.6	81.5	81.4	82.2	80.1	0.03
Total fibre intake^b^	19.1	19.9	20.0	20.1	20.9	< .0001
Glycaemic index	50.7	52.1	52.1	52.6	52.6	< .0001
Glycaemic load	78.7	88.8	91.8	96.6	99.3	< .0001
Fruit and vegetable intake^b,d^	0.29	0.26	0.25	0.23	0.22	< .0001
Wholegrain intake^b,d^	0.09	0.08	0.08	0.07	0.07	< .0001

*p* values were calculated using analysis of variance (ANOVA)

^a^Kilojoules/day

^b^Grams/day

^c^Milligrams/day

^d^% total dietary intake

Women in quintile 5 compared to those in quintile 1 had the highest intake of total energy, carbohydrates, sugars, sodium, glycaemic index, and glycaemic load. Those in quintile 5 also had higher intake of fat and different types of fats, including total fat, monounsaturated fat, polyunsaturated and saturated fat compared to those in quintile 1, but the lowest intake of cholesterol. Women in quintile 1 of UPF proportional intake had the highest intake of fruit and vegetables, and wholegrains compared to those in quintile 5), but the lowest total protein intake.

### Primary and secondary event outcomes

During the 15-year follow-up, there were 1,038 (10.8%) incident CVD cases and 471 (4.7%) deaths due to any cause. There were 4,204 (43.8%) cases of hypertension, 1,219 (12.7%) cases of type 2 DM, and 3,596 (36.0%) cases of obesity.

### Association of UPF intake with CVD

There was no significant association between CVD and UPF intake (*p*_trend_ = 0.18). In our final multivariate-adjusted model (Model 3), there was no significant association between the highest (> 34.2% of total dietary intake) versus lowest (< 18.1% of total dietary intake) intake of UPF with incident CVD (OR 1.22, 95% CI 0.92–1.61, *p* = 0.16) (Table 3).

**Table 3:** Associations between ultra-processed food (g/d) and incident cardiovascular outcomes among middle aged women (n = 9591)

	Quintile 1	Quintile 2		Quintile 3		Quintile 4			Quintile 5		
	Reference	OR	95% CI, *p* value	OR	95% CI, *p* value	OR		95% CI, *p* value	OR	95% CI, *p* value	*P* trend
Primary endpoints											
CVD											
Univariate	1.0 (reference)	0.92	0.75–1.13, *p* = 0.44	1.08	0.88–1.31, *p* = 0.48	0.99		0.81–1.21, *p* = 0.91	1.04	0.85–1.27, *p* = 0.73	0.55
Model 1	1.0 (reference)	0.93	0.73–1.19, *p* = 0.57	1.26	1.00–1.59, ***p* = 0.05**	1.04		0.82–1.32, *p* = 0.76	1.15	0.91–1.46, *p* = 0.25	0.17
Model 2	1.0 (reference)	0.91	0.70–1.18, *p* = 0.49	1.18	0.92–1.51, *p* = 0.20	0.97		0.75–1.26, *p* = 0.82	1.17	0.91–1.51, *p* = 0.22	0.20
Model 3	1.0 (reference)	0.92	0.71–1.20, *p* = 0.56	1.21	0.93–1.56, *p* = 0.16	1.00		0.76–1.32, *p* = 1.00	1.22	0.92–1.61, *p* = 0.16	0.18
Secondary endpoints											
Hypertension											
Univariate	1.0 (reference)	1.14	0.97–1.35, *p* = 0.12	1.12	0.95–1.32, *p* = 0.16	1.14		0.97–1.34, *p* = 0.12	1.16	0.99–1.37, *p* = 0.07	0.11
Model 1	1.0 (reference)	1.18	0.97–1.43, *p* = 0.10	1.26	1.05–1.53, ***p* = 0.02**	1.26		1.04–1.53, ***p* = 0.02**	1.24	1.03–1.50, ***p* = 0.03**	**0.03**
Model 2	1.0 (reference)	1.22	0.99–1.50, *p* = 0.06	1.23	1.00–1.51, ***p* = 0.05**	1.28		1.04–1.58, ***p* = 0.02**	1.31	1.07–1.61, ***p* = 0.01**	**0.02**
Model 3	1.0 (reference)	1.26	1.02–1.55, ***p* = 0.04**	1.26	1.02–1.56, ***p* = 0.04**	1.32		1.06–1.65, ***p* = 0.01**	1.39	1.10–1.74, ***p* = 0.005**	**0.02**
All-cause mortality											
Univariate	1.0 (reference)	0.78	0.59–1.04, *p* = 0.09	0.73	0.55–0.98, ***p* = 0.04**	0.77		0.58–1.02, *p* = 0.07	0.81	0.61–1.07, *p* = 0.13	0.15
Model 1	1.0 (reference)	0.75	0.54–1.04, *p* = 0.09	0.72	0.52–1.01, *p* = 0.05	0.75		0.54–1.04, *p* = 0.08	0.76	0.55–1.06, *p* = 0.10	0.10
Model 2	1.0 (reference)	0.73	0.51–1.05, *p* = 0.09	0.79	0.56–1.13, *p* = 0.20	0.85		0.60–1.21, *p* = 0.37	0.79	0.55–1.14, *p* = 0.21	0.38
Model 3	1.0 (reference)	0.74	0.51–1.06, *p* = 0.10	0.81	0.56–1.17, *p* = 0.25	0.87		0.60–1.27 *p* = 0.48	0.80	0.54–1.20, *p* = 0.28	0.49
Secondary endpoints											
Type 2 diabetes mellitus											
Univariate	1.0 (reference)	1.25	1.00–1.57, ***p* = 0.05**	1.13	0.90–1.42, *p* = 0.28		1.34	1.07–1.66, ***p* = 0.01**	1.19	0.95–1.49, *p* = 0.13	0.11
Model 1	1.0 (reference)	1.24	0.96–1.61, *p* = 0.10	1.14	0.87–1.48, *p* = 0.34		1.25	0.97–1.62, *p* = 0.09	1.13	0.86–1.47, *p* = 0.38	0.48
Model 2	1.0 (reference)	1.30	0.98–1.74, *p* = 0.07	1.16	0.87–1.56, *p* = 0.31		1.16	0.86–1.55, *p* = 0.33	1.26	0.94–1.70, *p* = 0.13	0.32
Model 3	1.0 (reference)	1.25	0.93–1.68, *p* = 0.14	1.10	0.81–1.50, *p* = 0.53		1.08	0.79–1.48, *p* = 0.64	1.17	0.84–1.63, *p* = 0.35	0.74
Obesity											
Univariate	1.0 (reference)	1.12	0.92–1.36, *p* = 0.25	1.35	1.11–1.63, ***p* = 0.002**		1.12	0.92–1.36, *p* = 0.26	1.03	0.85–1.26, *p* = 0.74	0.81
Model 1	1.0 (reference)	1.10	0.88–1.38, *p* = 0.41	1.38	1.11–1.72, ***p* = 0.004**		1.11	0.88–1.38, *p* = 0.38	1.01	0.81–1.27, *p* = 0.91	0.86
Model 2	1.0 (reference)	1.02	0.77–1.36, *p* = 0.88	1.23	0.93–1.63, *p* = 0.15		0.98	0.74–1.31, *p* = 0.90	1.05	0.79–1.40, *p* = 0.75	0.96
Model 3	1.0 (reference)	1.07	0.80–1.43, *p* = 0.67	1.29	0.97–1.73, *p* = 0.08		1.05	0.77–1.43, *p* = 0.76	1.16	0.85–1.60, *p* = 0.36	0.52

Model 1 was adjusted for age, area of residence, marital status, occupation, country of birth, qualification, and household income

Model 2: Model 1 + adjustment for body mass index, menopausal status, type 2 diabetes mellitus, hypertension, physical activity levels, and smoking status

Model 3: Model 2 + adjustment for total fibre, total carbohydrate, total fat intake, total protein, total energy intake (kilojoules), and total alcohol

*CVD* cardiovascular disease, *OR* odds ratio, *CI* confidence intervals

*p* values that were considered significant (*p* ≤ 0.05) were in bold

### Association of UPF intake with secondary endpoints

Our multivariable analysis demonstrated a significant association between increasing UPF intake and hypertension (*p*_trend_ = 0.02) (Table 3). In the final multivariable model (Model 3), the OR for hypertension with the highest (Quintile 5) versus lowest quintile (Quintile 1) of UPF intake was 1.39 (95% CI 1.10–1.74, *p* = 0.005). Quintile 2 (OR 1.26, 95% CI 1.02–1.55, *p* = 0.04), quintile 3 (OR 1.26, 95% CI 1.02–1.56, *p* = 0.04) and quintile 4 (OR 1.32, 95% 1.06–1.65, *p* = 0.01) were all associated with higher odds of hypertension compared with the lowest UPF intake (quintile 1). There was no significant association between UPF intake and all-cause mortality (OR 0.80, 95% CI 0.54–1.20, *p* = 0.28; *p*_trend_ = 0.49), type 2 DM (OR 1.17, 95% CI 0.84–1.63, *p* = 0.35; *p*_trend_ = 0.74), or obesity (OR 1.16, 95% CI 0.85–1.60, *p* = 0.36; *p*_trend_ = 0.52) (Table 3).

The findings remained consistent for all sensitivity models (Online Resource Table 2).

## Discussion

In this large prospective cohort study of Australian women, we found that a higher versus lower intake of UPF was associated with increased odds of hypertension. The 39% increased odds of hypertension with higher versus lower UPF intake in women was significant after adjusting for socio-demographic, medical, and dietary confounders. Additionally, more than a quarter of an average Australian woman’s diet comprised UPF, and a diet high in UPF had higher amounts of total energy, sugars, fat, and sodium.

To our knowledge, this is the first prospective study that has investigated the relationship between UPF intake, CVD and/or incident hypertension, specifically in women. Our study included a large female cohort, selected to be nationally representative of middle-aged Australian women. We found that the highest percentage of UPF intake (> 34.2% of total dietary intake) was associated with 39% higher odds of hypertension. The association with hypertension was consistent with a recent 2023 meta-analysis [32] as well as previous prospective studies, including the ELSA-Brasil Study (23% increased incidence) [33] and SUN (21% increased incidence) [34]. Hypertension continues to be the most undertreated and underdiagnosed risk factor for CVD and mortality globally, and to reduce disease burden, dietary modification is important UPFs are major sources of excess dietary salt and are energy dense, high in added sugars and saturated fat, but low in fruit and vegetables, therefore limiting these foods within a healthy diet may help lower blood pressure [34].

Our results differed from prior prospective studies and meta-analyses that demonstrated a significant positive association between higher UPF intake and CVD and all-cause mortality [11–14, 16–19, 35–37]. In the current study, UPF intake was not associated with increased odds of incident CVD or all-cause mortality. Non-sex-specific analyses that previously reported a significant association with incident CVD included UK Biobank [11, 14], Framingham Heart Study [12], NutriNet-Sante [17], and Atherosclerosis Risk in Communities (ARIC) study [13], ranging from a 4% to 19% increased incident CVD. This is likely reflective of the smaller number of cardiovascular outcomes and deaths in this female population over a follow-up of 15 years (10.8% of CVD events and an all-cause mortality of 4.7%), contrary to previous studies [15] that showed rates as high as 21.5% [12] for CVD and 9.6% [19] for mortality. Likewise for type 2 DM and obesity, other cohort studies and meta-analyses found higher UPF intake associated with both type 2 DM (12% to 44% increased risk] and obesity (20% to 55% increased risk) [8, 38–43]. Our sample size may have been too small, and therefore underpowered to detect the true effect, given that women have lower event rates than men, despite our long 15-year follow-up. For example, one study showed that men still had a two-fold higher age-specific predicted myocardial infarction risk compared to women in ages 55–74 years [44]. Moreover, the conflicting results may be attributed to varying methodologies, e.g., outcome ascertainment, follow-up duration, and socio-demographic factors for example sex and age, that may associate with decreased UPF intake or reduce the number of overall deaths [45]. Differences in assessing UPF intake cannot be ruled, despite using the NOVA food classification system as did other studies. NOVA classification is a descriptive way of categorising UPF; FFQs do not always capture enough detail to adequately classify foods such as yoghurt or bread into the appropriate category [20, 46]. Regarding the lack of associations for UPF intake with CVD or mortality in this middle-aged Australian population, factors, such as healthcare utilisation and higher socio-economics status, could have greater influence than dietary factors. Although we adjusted for some socio-demographic factors, residual confounding cannot be ruled out and unmeasured socio-economic factors, such as stress, may be associated with poor clinical outcomes.

Our findings are important to understand the impact of diet on cardiovascular health in women and reinforce the need for sex-specific research and recommendations. The majority of early clinical trials on the management of cardiovascular risk factors, such as hypertension, have been predominantly men [47]. The few studies that reported sex-specific results on UPF intake and cardiovascular health show there is a significant effect in women [8, 18, 19]. Interestingly, some have suggested the effect of UPFs may be more significant in women than in men, while others have reported no significant sex interaction for all-cause mortality [18, 19] and incident CVD [14]. Similar to our study, Kityo et al. [20] found no significant associations in both sexes for all-cause mortality. In this Korean population, the lower mortality rates may have been influenced by the middle-aged demographic and nutritional profile with a low UPF intake of 25.1%, and therefore the insignificant associations [20]. Studies have also demonstrated that a higher UPF intake was more associated with hypertension, type 2 DM, and obesity for women compared to men [8–10]. Our study further confirms the association between UPF intake and hypertension in women. This adds to the need for sex-specific studies that focus on preventative ways to reduce hypertension-related burden and consequently decrease the risk of CVD, such as by limiting intake of UPFs.

Knowledge of the adverse health effects of UPFs and the underlying pathophysiology is important in clinician education and public health messaging. Adverse health effects associated with UPFs may be due to displacement of cardio-protective foods, combined with higher intake of sugars, fat, sodium, and additives (artificial sweeteners and emulsifiers) [7, 48]. Excessive refined sugar from UPF correlates with higher glycaemic index, which can contribute to insulin resistance and type 2 DM [7, 8] while high sodium increases risk of hypertension [9, 34, 49]. Dietary additives and lower fibre in UPFs can alter the gut microbiome and induce low-grade inflammation [7, 50, 51]. Finally, higher saturated and trans-unsaturated fat intake increases risk of dyslipidaemia and atherosclerotic disease by activating downstream inflammation [7]. These aspects can ultimately result in oxidative stress, endothelial dysfunction, and a pro-inflammatory atherogenic state, all relevant to CVD pathophysiology [7, 52]. Furthermore, UPFs are energy-dense, and designed to be palatable, leading to over-eating, weight gain and obesity [7, 10]. However, we found a persistent association between UPF intake and hypertension, after adjustment for dietary fibre, energy–density of the food, fat, and carbohydrate content [7, 48]. Therefore, other factors must be at play in the detrimental health effects of UPF. We know that fruit and vegetables, olive oil, legumes, nuts, whole-grains, and fish have cardio-protective effects through higher intake of antioxidants, polyphenols, and omega-3 fatty acids [7, 48].

In the current study, the association between hypertension and UPF intake remained significant despite adjusting for fibre intake and other key macronutrients, such as total fat and energy, suggesting that the association could be driven by the degree of processing, not just the macronutrient intake. However, UPF would likely still provide lower intakes of micronutrients than less processed foods. Our study also demonstrated that fibre intake increases as UPF intake increased. This may be due to the high consumption of whole-wheat or whole-grain products, often high in dietary fibre, such as bread and breakfast cereals. In the current study, these products were categorised as UPF, as a large majority consumed are still refined and industrially processed [53]. However, more studies need to be designed to confirm whether processing itself plays a role in these associations with increasing UPF intake.

### Limitations

The study is observational and although many confounders including socio-economic factors were accounted for, we cannot adjust for unmeasured factors associated the outcome. Outcomes were based on self-report and date of events was not collected, with possibility of misclassification bias and potential for missed events. Dietary data were self-reported, which may lead to under- or over-reporting of food items or recall bias. Missing baseline data may have impacted our ability to adjust for confounders, but this was reduced by performing multiple imputations. Further, while the missingness of outcome data was low (4.1% for CVD and 4.3% for Hypertension), we acknowledge the differences in baseline characteristics between participants with and without missing outcome data may have introduced attrition bias due to incomplete follow-up data. However, as the missingness was small, the biases would have little impact on the main results. The FFQ was not designed to separate food items into the NOVA classes. This may result in misclassification of food, for example, muesli and pizza were categorised as minimally processed and bread classified as ultra-processed, due to difficulty distinguishing minimally processed/home-made from industrially produced versions. Nevertheless, Australian nationally representative data was used for final categorisation (e.g., mostly non-UPF pizzas as consumed in Australia) [5]. Our study included only middle-aged Australian women and therefore cannot be generalizable to women of all age groups or origins, particularly as younger adults tend to have a diet higher in UPFs [54]. Dietary habits may have changed over time with our cohort having a lower UPF intake (26.6% of total dietary intake) compared to more recent surveys [5, 55, 56], however, mean UPF in our cohort of women was similar in the most recently available FFQ (2013), at 22.6%. Further, we excluded participants with previous history of CVD since a cardiovascular event before the dietary survey could have affected diet. This may lead to collider stratification bias within the studied sample, limiting generalisability. Finally, there is a possibility of competing risk with our main endpoints. For example, participants may have had less time to develop other outcomes, such as CVD, obesity, or DM, if UPF was associated with early mortality. However, this is an unlikely large source of bias as all-cause mortality differences were not detectable.

### Future implications

Our study expands upon previous research on UPFs and cardiovascular health, particularly incident hypertension. In Australia, current dietary advice recommends limiting intake of foods that are high in sodium, sugars, and fat, however, specific recommendations regarding UPF intake has not entered nutritional guidelines. Future clinical trials are needed to test the efficacy of limiting UPFs in women for prevention of hypertension and CVD and whether the degree of processing alone is sufficient to account for these associations.

## Conclusions

Our large cohort of women demonstrated that higher UPF intake was significantly associated with increased incidence of hypertension, with no effect on incident CVD which may be limited by the smaller number of CVD events. Our study reinforces the importance of sex-specific analyses that focus on the dietary intake of women.

### Supplementary Information

Below is the link to the electronic supplementary material.

Supplementary file1 (DOCX 43 KB)

## Data Availability

Datasets analysed in this study may be obtained with approval from the third party (ALSWH) and are not publicly available.

## Abbreviations

CVD: Cardiovascular disease
UPF: Ultra-processed food
DM: Diabetes mellitus
ALSWH: Australian Longitudinal Study on Women’s Health
FFQ: Food Frequency Questionnaire
BMI: Body Mass Index
MBS: Medicare benefits schedule
PBS: Pharmaceutical benefits scheme
NDI: National death index
SD: Standard deviation
