# Supplementary material for: Ultra-processed foods and incident cardiovascular disease and hypertension in middle-aged women
Source: Eur J Nutr. 2023 Dec 26;63(3):713–25. doi: 10.1007/s00394-023-03297-4 (PMC10948520; doi:10.1007/s00394-023-03297-4)
Supplement: Supplementary file 1 — Supplementary file1 (DOCX 43 KB) [file 394_2023_3297_MOESM1_ESM.docx]

**SUPPLEMENTARY MATERIALS**

**Ultra-processed Foods and Incident Cardiovascular Disease and Hypertension in Women**

**Brief Title: Pant A, et al. Ultra-processed Food and CVD in Women.**

**Author Names and Academic Degrees:** Anushriya Pant,^a^ BMedSc, Sarah Gribbin ^b^ BMedSc, MD, Priscila Machado,^c^ PhD, Allison Hodge,^d,e^ BSc, GDip(Diet), PhD, Simone Marschner,^a^ MSc, Jason H. Wasfy,^g^ MD, MPhil, Lisa Moran,^h^ BSc, BND, PhD, Clara K Chow,^a^ MBBS, PhD, Sarah Zaman,^a,f^ MBBS, PhD.

**Author Affiliations:**

^a^ Westmead Applied Research Centre and Faculty of Medicine and Health, University of Sydney, Westmead, New South Wales, Australia

^b^ Department of General Medicine, The Alfred Hospital, Alfred Health, Melbourne, Victoria, Australia

^c^ Institute for Physical Activity and Nutrition, School of Exercise and Nutrition Sciences, Deakin University, Melbourne, Victoria, Australia

^d^ Cancer Epidemiology Division, Cancer Council Victoria, Melbourne, Victoria, Australia.

^e^ Centre for Epidemiology and Biostatistics, Melbourne School of Population and Global Health, The University of Melbourne, Parkville, Victoria, Australia.

^f^ Department of Cardiology, Westmead Hospital, Westmead, New South Wales, Australia

^g^ Cardiology Division, Massachusetts General Hospital and Harvard Medical School, Boston, Massachusetts, United States of America

^h^ Monash Centre for Health Research and Implementation, Monash University, Melbourne, Victoria, Australia.

**Corresponding author:** Associate Professor Sarah Zaman

Westmead Applied Research Centre, The University of Sydney, Wesmtead, New South Wales, Australia, 2145; Email: [sarah.zaman@sydney.edu.au](mailto:sarah.zaman@sydney.edu.au)

**Supplementary Table 1.** Groups and subgroups of the NOVA classification applied to the food frequency questionnaire (FFQ) of the ALSWH.

| **Food item** | **Food subgroup** | **NOVA group** | **UPF (1)/ non-UPF (0)** |
| --- | --- | --- | --- |
| Full cream milk | Milk and plain yoghurt | 1 | 0 |
| Reduced fat milk | Milk and plain yoghurt | 1 | 0 |
| Skim milk | Milk and plain yoghurt | 1 | 0 |
| Soya milk | Milk-based drinks | 4 | 1 |
| Bread White bread Hi-fibre | Mass-produced packaged breads | 4 | 1 |
| Bread White bread | Mass-produced packaged breads | 4 | 1 |
| Bread Wholemeal bread | Mass-produced packaged breads | 4 | 1 |
| Bread rye bread | Mass-produced packaged breads | 4 | 1 |
| Multigrain bread | Mass-produced packaged breads | 4 | 1 |
| Margarine | Margarine and other spreads | 4 | 1 |
| Polyunsaturated margarine | Margarine and other spreads | 4 | 1 |
| Monounsaturated margarine | Margarine and other spreads | 4 | 1 |
| Butter and margarine blend | Margarine and other spreads | 4 | 1 |
| Butter | Animal fats | 2 | 0 |
| Hard cheese | Cheese | 3 | 0 |
| Firm cheese | Cheese | 3 | 0 |
| Soft cheese | Cheese | 3 | 0 |
| Ricotta or cottage cheese | Cheese | 3 | 0 |
| cream cheese | Cheese | 3 | 0 |
| Low fat cheese | Cheese | 3 | 0 |

**Supplementary Table 1.** Continued.

| **Food item** | **Food subgroup** | **NOVA group** | **UPF (1)/ non-UPF (0)** |
| --- | --- | --- | --- |
| Sugar | Other processed culinary ingredients | 2 | 0 |
| Eggs | Eggs | 1 | 0 |
| All Bran | Breakfast cereals | 4 | 1 |
| Sultana Bran™, FibrePlus™, Branflakes™ | Breakfast cereals | 4 | 1 |
| Weet Bix™, Vita Brits™, Weeties™ | Breakfast cereals | 4 | 1 |
| Cornflakes, Nutrigrain™, Special K™ | Breakfast cereals | 4 | 1 |
| Porridge | Cereals | 1 | 0 |
| Muesli | Cereals | 1 | 0 |
| Rice | Cereals | 1 | 0 |
| Pasta or noodles | Pasta | 1 | 0 |
| Crackers, crispbreads, dry biscuits | Packaged salty snacks | 4 | 1 |
| Sweet biscuits | Biscuits | 4 | 1 |
| Cakes, sweet pies, tarts and other sweet pastries | Pastries, buns, and cakes | 4 | 1 |
| Meat pies, pastries, quiche and other savoury pastries | Packaged ready meals | 4 | 1 |
| Pizza | Pasta | 1 | 0 |
| Hamburger with a bun | Packaged ready meals | 4 | 1 |
| Chocolate | Confectionary | 4 | 1 |
| Flavoured milk drink (coca, Milo (TM) etc) | Milk-based drinks | 4 | 1 |
| Nuts | Nuts and seeds | 1 | 0 |

**Supplementary Table 1.** Continued.

| **Food item** | **Food subgroup** | **NOVA group** | **UPF (1)/ non-UPF (0)** |
| --- | --- | --- | --- |
| Peanut butter or peanut paste | Legumes | 1 | 0 |
| Corn chips, potato crisps, Twisties(TM) etc | Packaged salty snacks | 4 | 1 |
| Jam, marmalade, honey or syrups | Other processed foods | 3 | 0 |
| Vegemite(TM), Marmite(TM) or Promite™ | Margarine and other spreads | 4 | 1 |
| Ice-cream | Ice cream, ice pops and frozen yogurts | 4 | 1 |
| Yoghurt | Milk-based drinks | 4 | 1 |
| Beef | Meat | 1 | 0 |
| Veal | Meat | 1 | 0 |
| Chicken | Poultry | 1 | 0 |
| Lamb | Meat | 1 | 0 |
| Pork | Meat | 1 | 0 |
| Bacon | Reconstituted meat products | 4 | 1 |
| Ham | Reconstituted meat products | 4 | 1 |
| Corned beef, luncheon meats or salami | Reconstituted meat products | 4 | 1 |
| Sausages or frankfurters | Reconstituted meat products | 4 | 1 |
| Fish (steamed, grilled or baked) | Fish | 1 | 0 |
| Fish (fried) | Fish | 1 | 0 |
| Fish (tinned) | Bacon and other salted, smoked or canned meat or fish | 3 | 0 |
| Fruit (tinned or frozen fruit) | Fruit | 1 | 0 |

**Supplementary Table 1.** Continued.

| **Food item** | **Food subgroup** | **NOVA group** | **UPF (1)/ non-UPF (0)** |
| --- | --- | --- | --- |
| Fruit juice | Packaged ready meals | 4 | 1 |
| Oranges or other citrus fruits | Fruit | 1 | 0 |
| Apples | Fruit | 1 | 0 |
| Pears | Fruit | 1 | 0 |
| Bananas | Fruit | 1 | 0 |
| Watermelon, rockmelon (cantaloupe), honeydew etc | Fruit | 1 | 0 |
| Pineapple | Fruit | 1 | 0 |
| Strawberries | Fruit | 1 | 0 |
| Apricots | Fruit | 1 | 0 |
| Peaches or nectarines | Fruit | 1 | 0 |
| Mango or paw paw | Fruit | 1 | 0 |
| Avocado | Fruit | 1 | 0 |
| Potatoes roasted or fried (include hot chips) | Industrial potato chips | 4 | 1 |
| Potatoes cooked without fat | Potato | 1 | 0 |
| Tomato sauce, tomato paste | Sauces, dressing and gravies | 4 | 1 |
| Fresh or tinned tomatoes | Vegetables | 1 | 0 |
| Peppers (capsicum) | Vegetables | 1 | 0 |
| Lettuce, endive, or other salad greens | Vegetables | 1 | 0 |
| Cucumber | Vegetables | 1 | 0 |
| Celery | Vegetables | 1 | 0 |
| Beetroot | Vegetables | 1 | 0 |

**Supplementary Table 1.** Continued.

| **Food item** | **Food subgroup** | **NOVA group** | **UPF (1)/ non-UPF (0)** |
| --- | --- | --- | --- |
| Carrots | Vegetables | 1 | 0 |
| Cabbage | Vegetables | 1 | 0 |
| Cauliflower | Vegetables | 1 | 0 |
| Broccoli | Vegetables | 1 | 0 |
| Silverbeet or spinach | Vegetables | 1 | 0 |
| Peas | Vegetables | 1 | 0 |
| Green beans | Vegetables | 1 | 0 |
| Bean sprouts or alfalfa sprouts | Vegetables | 1 | 0 |
| Baked beans | Packaged ready meals | 4 | 1 |
| Soy beans, soy bean curd or tofu | Legumes | 1 | 0 |
| Other beans (include chick peas, lentils etc) | Legumes | 1 | 0 |
| Pumpkin | Vegetables | 1 | 0 |
| Onion or leeks | Vegetables | 1 | 0 |
| Garlic (not garlic tablets) | Vegetables | 1 | 0 |
| Mushrooms | Vegetables | 1 | 0 |
| Zucchini | Vegetables | 1 | 0 |

*UPF*, ultra-processed food;

*NOVA classification system* was developed by Monteiro et al and categorises food according to four main groups based on the level of food process: 1) Unprocessed/Minimally-processed foods. These are foods that have are either edible parts of the plants or from animals (fat, eggs, milk etc) or altered by industrial procedures to remove inedible parts or extend shelf life; 2) Processed culinary ingredients. Food items that are utilised to cook and season group 1 foods and can be retrieved from nature either by extracting, refining, or other industrial processes; 3) Processed foods. These foods are made by adding group 2 items (sugar, salt, soil etc) to group 1 foods and preservation methods are used here to increase durability and taste; 4) Ultra-processed foods. These foods are designed using extensive, many industrial processes and ingredients like additives, hydrogenated oils, flavours, sweeteners, and emulsifiers.

**Supplementary Table 2.** Associations between ultra-processed food (g/d) and incident cardiovascular outcomes among middle aged women (Sensitivity analysis, multiple imputations).

|  | **Quintile 1** | **Quintile 2** | | **Quintile 3** | | **Quintile 4** | | **Quintile 5** | |  |
| --- | --- | --- | --- | --- | --- | --- | --- | --- | --- | --- |
|  | **Reference** | **OR** | **95% CI,**  **p-value** | **OR** | **95% CI,**  **p-value** | **OR** | **95% CI,**  **p-value** | **OR** | **95% CI,**  **p-value** | **P trend** |
| **Primary endpoints** | | | | | | | | | |  |
| **CVD** | | | | | | | | | |  |
| Univariate | 1.0 (reference) | 0.92 | 0.75 to 1.13, p=0.44 | 1.08 | 0.88 to 1.31, p=0.48 | 0.99 | 0.81 to 1.21, p=0.91 | 1.04 | 0.85 to 1.27, p=0.73 | 0.55 |
| Model 1 | 1.0 (reference) | 0.90 | 0.73 to 1.12, p=0.34 | 1.11 | 0.90 to 1.36, p=0.33 | 0.98 | 0.79 to 1.20, p=0.82 | 1.03 | 0.84 to 1.27, p=0.78 | 0.55 |
| Model 2 | 1.0 (reference) | 0.87 | 0.69 to 1.08, p=0.20 | 1.06 | 0.86 to 1.31, p=0.59 | 0.94 | 0.76 to 1.17, p=0.58 | 1.04 | 0.84 to 1.29, p=0.72 | 0.52 |
| Model 3 | 1.0 (reference) | 0.88 | 0.70 to 1.10, p=0.26 | 1.09 | 0.87 to 1.36, p=0.46 | 0.97 | 0.77 to 1.23, p=0.81 | 1.09 | 0.86 to 1.38, p=0.48 | 0.35 |
| **Secondary endpoints** | | | | | | | | | |  |
| **Hypertension** | | | | | | | | | |  |
| Univariate | 1.0 (reference) | 1.14 | 0.97 to 1.35, p=0.12 | 1.12 | 0.95 to 1.32, p=0.16 | 1.14 | 0.97 to 1.34, p=0.12 | 1.16 | 0.99 to 1.37, p=0.07 | 0.11 |
| Model 1 | 1.0 (reference) | 1.11 | 0.94 to 1.32, p=0.22 | 1.14 | 0.97 to 1.35, p=0.12 | 1.13 | 0.96 to 1.34, p=0.14 | 1.15 | 0.98 to 1.36, p=0.10 | 0.12 |
| Model 2 | 1.0 (reference) | 1.12 | 0.94 to 1.34, p=0.21 | 1.10 | 0.92 to 1.32, p=0.28 | 1.12 | 0.94 to 1.34, p=0.21 | 1.22 | 1.02 to 1.45, **p=0.03** | 0.06 |
| Model 3 | 1.0 (reference) | 1.14 | 0.95 to 1.37, p=0.17 | 1.12 | 0.93 to 1.34, p=0.25 | 1.14 | 0.94 to 1.38, p=0.20 | 1.25 | 1.03 to 1.53, **p=0.02** | 0.06 |

**Supplementary Table 2.** Continued.

|  | **Quintile 1** | **Quintile 2** | | **Quintile 3** | | **Quintile 4** | | **Quintile 5** | |  |
| --- | --- | --- | --- | --- | --- | --- | --- | --- | --- | --- |
|  | **Reference** | **OR** | **95% CI,**  **p-value** | **OR** | **95% CI,**  **p-value** | **OR** | **95% CI,**  **p-value** | **OR** | **95% CI,**  **p-value** | **P trend** |
| **Secondary endpoints** | | | | | | | | | | |
| **All-cause mortality** | | | | | | | | | |  |
| Univariate | 1.0 (reference) | 0.78 | 0.59 to 1.04, p=0.09 | 0.73 | 0.55 to 0.98, **p=0.04** | 0.77 | 0.58 to 1.02, p=0.07 | 0.81 | 0.61 to 1.07, p=0.13 | 0.15 |
| Model 1 | 1.0 (reference) | 0.82 | 0.61 to 1.09, p=0.18 | 0.77 | 0.57 to 1.03, p=0.08 | 0.81 | 0.60 to 1.08, p=0.14 | 0.85 | 0.64 to 1.13, p=0.26 | 0.21 |
| Model 2 | 1.0 (reference) | 0.87 | 0.64 to 1.18, p=0.37 | 0.84 | 0.62 to 1.15, p=0.28 | 0.95 | 0.70 to 1.29, p=0.73 | 0.95 | 0.70 to 1.30, p=0.76 | 0.81 |
| Model 3 | 1.0 (reference) | 0.87 | 0.64 to 1.19, p=0.38 | 0.84 | 0.61 to 1.17, p=0.30 | 0.95 | 0.68 to 1.32, p=0.77 | 0.95 | 0.68 to 1.34, p=0.79 | 0.85 |
| **Type 2 diabetes mellitus** | | | | | | | | | |  |
| Univariate | 1.0 (reference) | 1.25 | 1.00 to 1.57, **p=0.05** | 1.13 | 0.90 to 1.42, p=0.28 | 1.34 | 1.07 to 1.66, **p=0.01** | 1.19 | 0.95 to 1.49, p=0.13 | 0.11 |
| Model 1 | 1.0 (reference) | 1.26 | 1.00 to 1.59, **p=0.05** | 1.15 | 0.91 to 1.46, p=0.24 | 1.35 | 1.08 to 1.69, **p=0.01** | 1.20 | 0.95 to 1.51, p=0.12 | 0.12 |
| Model 2 | 1.0 (reference) | 1.31 | 1.02 to 1.68, **p=0.04** | 1.12 | 0.87 to 1.45, p=0.39 | 1.31 | 1.02 to 1.68, **p=0.04** | 1.28 | 0.99 to 1.65, p=0.06 | 0.10 |
| Model 3 | 1.0 (reference) | 1.26 | 0.97 to 1.62, p=0.08 | 1.07 | 0.82 to 1.39, p=0.64 | 1.22 | 0.94 to 1.60, p=0.14 | 1.20 | 0.90 to 1.58, p=0.22 | 0.40 |

**Supplementary Table 2.** Continued.

|  | **Quintile 1** | **Quintile 2** | | **Quintile 3** | | **Quintile 4** | | **Quintile 5** | |  |
| --- | --- | --- | --- | --- | --- | --- | --- | --- | --- | --- |
|  | **Reference** | **OR** | **95% CI,**  **p-value** | **OR** | **95% CI,**  **p-value** | **OR** | **95% CI,**  **p-value** | **OR** | **95% CI,**  **p-value** | **P trend** |
| **Secondary endpoints** | | | | | | | | | |  |
| **Obesity** | | | | | | | | | |  |
| Univariate | 1.0 (reference) | 1.12 | 0.92 to 1.36, p=0.25 | 1.35 | 1.11 to 1.63, **p=0.002** | 1.12 | 0.92 to 1.36, p=0.26 | 1.03 | 0.85 to 1.26, p=0.74 | 0.81 |
| Model 1 | 1.0 (reference) | 1.10 | 0.91 to 1.35, p=0.33 | 1.34 | 1.11 to 1.63, **p=0.003** | 1.10 | 0.90 to 1.34, p=0.37 | 1.00 | 0.82 to 1.22, p=1.00 | 0.99 |
| Model 2 | 1.0 (reference) | 1.01 | 0.78 to 1.29, p=0.96 | 1.28 | 1.00 to 1.64, **p=0.05** | 1.06 | 0.82 to 1.36, p=0.68 | 1.07 | 0.83 to 1.38, p=0.59 | 0.58 |
| Model 3 | 1.0 (reference) | 1.04 | 0.80 to 1.34, p=0.78 | 1.33 | 1.03 to 1.72, **p=0.03** | 1.12 | 0.86 to 1.47, p=0.41 | 1.17 | 0.88 to 1.54, p=0.28 | 0.27 |
| Model 1 was adjusted for age, area of residence, marital status, occupation, country of birth, qualification, and household income.  Model 2: Model 1 + adjustment for body mass index, menopausal status, type 2 diabetes mellitus, hypertension, physical activity levels, and smoking status.  Model 3: Model 2 + adjustment for fibre, % carbohydrate, % total fat intake, % protein, total energy intake (kilojoules/day), and % alcohol.  *CVD,* cardiovascular disease; *OR*, odds ratio; *CI,* confidence intervals.  P values that were considered significant (p≤0.05) were in bold. | | | | | | | | | | |
